# Dosimetric Challenges of Small Lung Lesions in Low-Density Tissue Treated with Stereotactic Body Radiation Therapy

**DOI:** 10.3390/jcm15020603

**Published:** 2026-01-12

**Authors:** Indra J. Das, Meisong Ding, Mohamed E. Abazeed

**Affiliations:** Department of Radiation Oncology, Northwest Memorial Hospital, Northwestern University Feinberg School of Medicine, Chicago, IL 60611, USA; meisong.ding@nm.org (M.D.); mohamed.abazeed@nm.org (M.E.A.)

**Keywords:** radiation treatment, inhomogeneity correction, heterotopic ossification, radiobiological consequence

## Abstract

**Background/Objectives:** Stereotactic body radiation therapy (SBRT) is widely used for small lung tumors, but the physics of electron transport in low-density lungs remains incompletely understood. This study quantifies the effect of lung density on dosimetry for small lesions. **Methods:** To study the dosimetric parameters a pseudo patient option was chosen. A lung SBRT patient with a central lesion was modeled in the Eclipse treatment planning system using the AAA algorithm. Three target sizes (1.0, 1.5, and 2.0 cm) were planned with lung densities overridden from 0.1 to 1.0 g/cm^3^. Standard SBRT constraints were applied, and dosimetry indices (CI, HI, GI), maximum dose, and MU/Gy were recorded to see the pattern. **Results:** Dose–volume histograms (DVHs) showed marked dependence on both lesion size and lung density. Lower densities produced higher maximum doses (up to 135% at 0.1 g/cm^3^), steeper DVH tails, and significantly increased MU/Gy. Conformity was achievable in all cases, but at the cost of degraded homogeneity and gradient indices. At higher density (1.0 g/cm^3^), maximum dose values fell to 108–110% which is typical in non-lung cases. **Conclusions:** SBRT planning in low-density lungs requires substantially higher MU and results in greater dose spillage despite acceptable conformity. These findings highlight the importance of considering density effects when comparing clinical outcomes across institutions and selecting optimal plans, where minimizing MU/Gy may reduce unnecessary dose burden.

## 1. Introduction

Early detection through low-dose CT (LDCT) screening has shifted the lung cancer landscape, with nearly 70% of detected tumors now in stage I, which is associated with improved outcomes [1,2,3,4]. For these small, early-stage lesions, stereotactic body radiation therapy (SBRT), also known as stereotactic ablative radiotherapy (SABR), has become the cornerstone of treatment for patients who are medically inoperable or refuse surgery, offering high local control with favorable cost-effectiveness [5].

**Figure 1:**
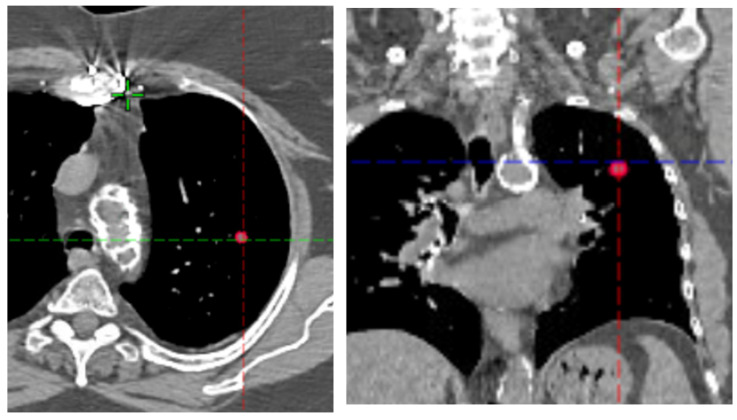
A small lesion (1.0 cm diameter PTV) in the center of the left lung. This volume poses significant challenge for dosimetry, representing similarity of air tissue buildup phenomenon for megavoltage photon beams.

Modern SBRT is delivered with megavoltage photon beams, which provide skin sparing but also exhibit dose buildup when transitioning from air to tissue. In the lung, where density varies widely due to disease state and breathing conditions (e.g., free breathing, deep inspiration breath hold, gating), this buildup effect is particularly pronounced. Reported lung densities range from −600 to −900 Hounsfield Units (HU), approximating near-air conditions. Small targets located centrally within lung tissue therefore present unique dosimetric challenges (Figure 1).

The physics of radiation transport in low-density media is a complex problem. Since Batho’s seminal work in 1964 [6], heterogeneity corrections have been refined with advances in computing power and dose calculation algorithms, culminating in AAPM Report 85 [7] which provided valuable data and recommendations for inhomogeneity correction. Monte Carlo studies have demonstrated superior accuracy in low-density settings [8,9], and algorithmic improvements have been incorporated into modern treatment planning systems [10,11]. Still, challenges remain for small field dosimetry in heterogeneous lungs and small target volumes that may lie within buildup regions.

Small fields used in SBRT/SABR with modulated delivery techniques such as VMAT introduce additional challenges in low-density media. Prior work by Das et al. [12] described the issue of source occlusion, which occurs when collimator jaws or multileaf collimators (MLCs) define very small fields that partially block the primary photon source. This occlusion reduces central-axis output and alters beam characteristics. While small-field dosimetry in homogeneous media has been comprehensively addressed in two special reports [13,14], comparable characterization in heterogeneous medium like lung tissue remains lacking, and its implication in SBRT is not fully understood.

In low-density lungs, lateral electron disequilibrium, already a recognized issue in homogeneous media per TG-155 [14], is further exaggerated, reducing dose deposition within the target. These effects make it intuitive that target coverage in SBRT is strongly dependent not only on tumor size and margin selection but also on underlying lung density, with a paucity of data. Multi-institutional studies have highlighted variability in SBRT dose prescription and reporting, reflecting wide dosimetric variation in target volume and monitor unit (MU) requirements for small lung lesions [15]. However, the underlying causes, particularly the interplay between target size and lung density, affecting electron transport, are not fully understood. It is speculated that buildup phenomenon associated with electron transport may be at play.

To study the dosimetric effect in lung SBRT, a pseudo patient was created where lung density and target size were changed. Since the topic is relevant to modern radiotherapy and is an area that deserves additional research, since many clinics are benchmarking lung SBRT plan quality based on clinical trial guidelines that are now decades old, a large variation in dosimetry is observed [11]. Much has changed since then in terms of delivery techniques, optimization algorithms, and dose calculations algorithms in addition to our understanding that PTV size alone is not the only predictor of plan quality.

This study investigates the dosimetric impact of lung density on small targets treated with SBRT, focusing on dose buildup phenomena, DVH tail behavior, and MU requirements. By quantifying these effects, we aim to provide data between conformity, dose spillage, and planning efficiency in terms of MU in low-density lungs and small target volume.

## 2. Materials and Methods

Under institutional review board (IRB) approval, one representative patient with a central lung lesion (Figure 1) was selected. Treatment planning was performed in the Eclipse treatment planning system (Varian Medical System, Palo Alto, CA, USA) Version 16.1 with the anisotropic analytical algorithm (AAA), with 6 MV beam from TrueBeam, where gross tumor volumes (GTVs) and planning target volumes (PTVs) were defined. Three clinically relevant PTV sizes were modeled, 1.0, 1.5, and 2.0 cm in diameter, to represent sizes used clinically in SBRT and relevant to the aim of this study.

To simulate varying lung conditions, the physical density of the lung was systematically overridden to values of 0.1, 0.2, 0.3, 0.5, 0.8, and 1.0 g/cm^3^, corresponding to Hounsfield Unit (HU) ranges from approximately −600 to near water-equivalent tissue. Densities above 0.3 g/cm^3^ were included to represent select patient scenarios, while the 1.0 g/cm^3^ setting reflected the extreme case of “no inhomogeneity correction,” which is still in use in some clinical trials and institutions in many parts of the world and correlates with non-lung patient diseases. With six densities and three volumes, a total of 18 plans were studied.

**Figure 2:**
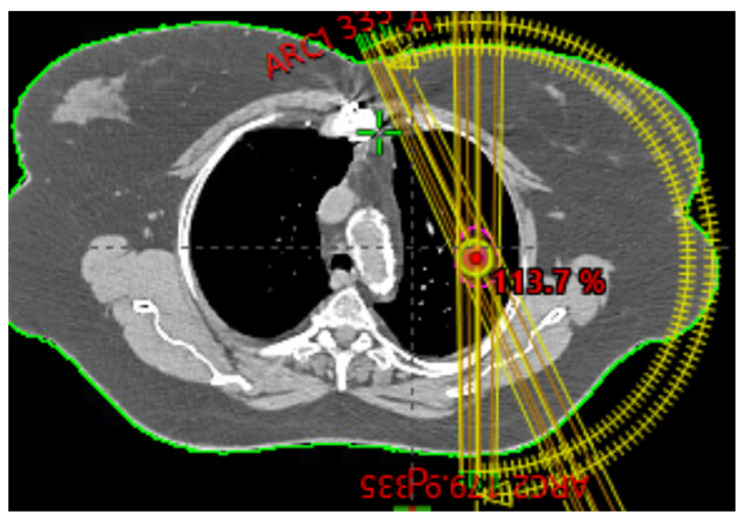
Beam arrangements of two partial ipsilateral arcs for lung SBRT.

Organs at risk (OARs) from the original CT dataset (e.g., bronchial structures, heart, lungs, esophagus, liver) were contoured and evaluated against published SBRT dose–volume constraints [16,17,18]. SBRT plans were generated to deliver 54 Gy in three fractions (18 Gy/fraction) using 6 MV photons, with identical OAR constraints applied across all cases. Two partial ipsilateral arcs, which are traditionally used for patient treatment as shown in Figure 2, were used in this study for all plans. These are copied from the clinical cases used in our clinic. The photon optimization setting, the convergence setting, and aperture shape controller options were set to OFF, which is typically clinically performed. To avoid initial bias, each case was optimized from a new start with the same identical constraints for all 18 cases.

Our machines are calibrated in reference condition for a source-to-surface distance (SSD) of 100 cm at d_max_. This is relevant for analysis of MU with respect to lung density and target size.

Dose calculations were performed in Eclipse using the AAA. Although Acuros and AAA are known to differ by 3–5% in heterogeneous media [18,19], only AAA was employed in this study for consistency as it is used in our clinical setting. A separate study is underway to compare our results with another algorithm and with the newer version 18.1, which is being validated. For each plan, dose–volume histogram (DVH) data were extracted, including D_100_, D_98_, D_50_, D_2_, and tail-end parameters. Standard dosimetric indices were calculated: conformity index (CI), homogeneity index (HI), and gradient index (GI) [20,21]. While HI is sometimes debated in lung SBRT due to heterogeneous tissue, it was included here to provide a measure of DVH steepness. GI, routinely used in SBRT and SRS, quantifies dose spillage from the target volume.

In addition, the maximum monitor units (MU) were recorded, as MU correlates with integral dose and treatment machine workload. Although alternative indices such as R_50_ have been proposed [22,23], this study adhered to international guideline definitions [20,24], as summarized in the following equations:

(1) $$CI=\frac{V_{RX}}{V_{PTV}}$$

(2) $$HI=\frac{\left(D_{2}-D_{98}\right)}{D_{50}}$$

(3) $$GI=\frac{V_{50\%}}{V_{100\%}}$$

## 3. Results

A total of 18 plans (3 lesion sizes × 6 lung densities) were analyzed. Despite identical DVH constraints, monitor unit (MU) requirements varied between iterations due to the stochastic nature of the optimization routine in the Eclipse system [25]. While CI, HI, and GI remained consistent, MU differed by up to ±9.6% across repeated plans, as reflected in the error bars shown in subsequent Figures.

**Figure 3:**
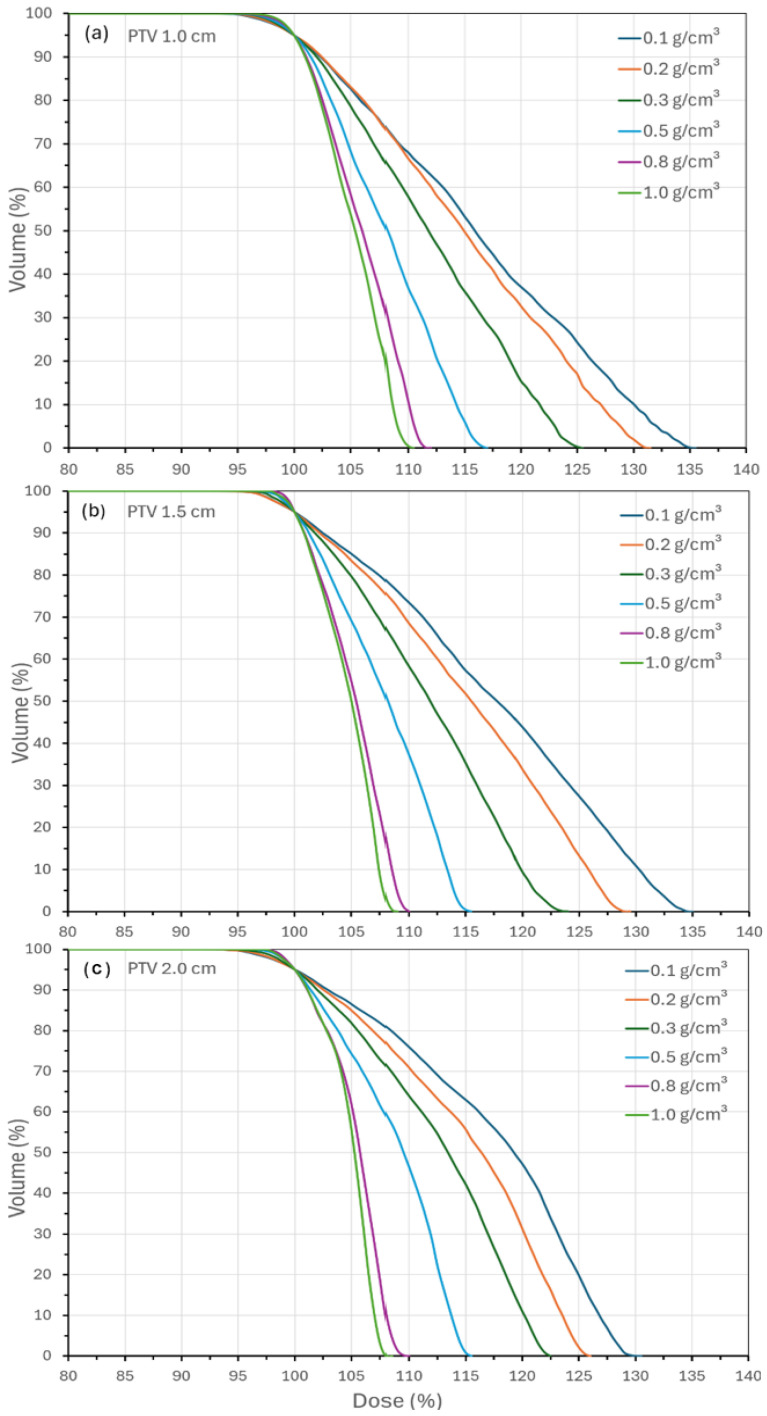
DVH data for three PTV sizes showing long tailed curve for low lung density, (**a**) 1.0 cm, (**b**) 1.5 cm, and (**c**) 2 cm diameter PTV.

Figure 3 presents PTV DVHs for the three lesion sizes (1.0, 1.5, and 2.0 cm) across the six lung densities. As density decreased, electron range increased, producing flatter DVHs and higher maximum doses, with hotspots reaching 135% at 0.1 g/cm^3^. In typical clinical settings, hotspots are limited to within 7–10%, but in low-density lungs this is not achievable. The effect is compounded by smaller PTVs, where the buildup depth of 6 MV photons (≈1.5 cm) is not met, leading to unavoidable spillage. At a higher density (1.0 g/cm^3^), DVH tails converged toward values more typical of other disease sites, with maximum doses of 110%, 109%, and 108% for the 1.0, 1.5, and 2.0 cm PTVs, respectively.

**Figure 4:**
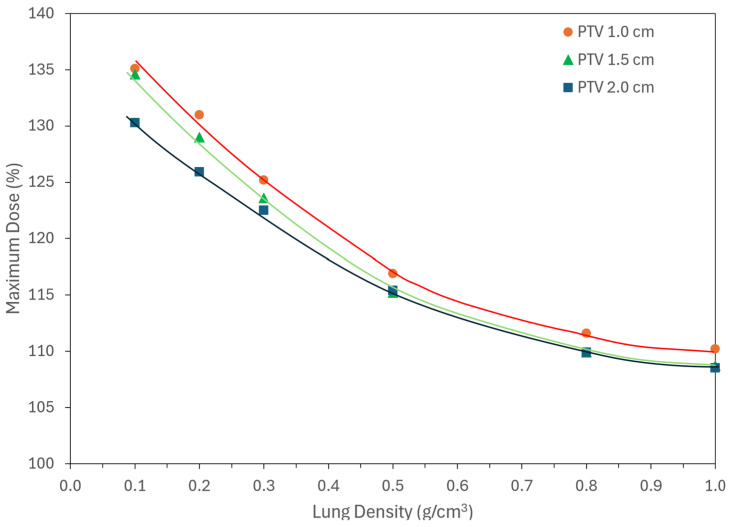
Maximum dose as a function of lung density for three PTV diameters (1.0, 1.5, and 2.0 cm). Lower lung densities produce substantially higher maximum doses due to increased electron range and dose spillage, with the effect most pronounced for smaller lesions. Lines are drawn to show the trend and not reflect any curve fitting.

Figure 4 plots the maximum dose as shown in the tail end of the DVH in Figure 3 against the lung density for the same three lesions. Descriptions of maximum dose have evolved over time. It used to be described as a maximum dose received in 1.0 cm^3^ volume [26] and was later revised to D_2_ in a modulated beam (IMRT, VMAT, SBRT) in ICRU-83 [20]. However, most treatment planning still provides it as a maximum dose point (as shown in Figure 4). As expected, decreasing lung density markedly increased maximum dose due to enhanced electron range and dose spillage. The effect is relatively less pronounced in large lesions. The maximum dose is traditionally tolerated within ±10%, but for lung SBRT in low density it is ~3-fold higher. For 1.0, 1.5, and 2 cm PTV, the maximum was 135%, 134%, and 130%, respectively, for 0.1 g/cm^3^ density. However, as the density increased to 1.0 g/cm^3^, the magnitude reduced to 110%. 109%, and 108%, respectively. The magnitude of maximum point for three curves is nearly the same, but it does change significantly based on the density of the medium.

**Figure 5:**
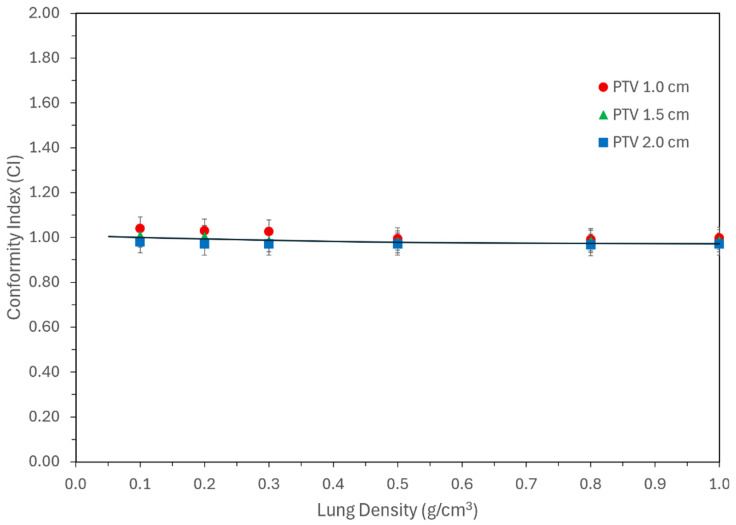
Conformity index (CI) as a function of lung density for three PTV diameters (1.0, 1.5, and 2.0 cm). CI remained near 1.0 across all conditions, demonstrating consistent target coverage regardless of density or target size.

Figure 5 presents CI values as a function of lung density for the three PTV sizes. It shows that CI remained consistently close to 1.0 across all densities and lesion sizes, indicating that adequate target coverage was achieved in every case. Notably, there was no meaningful variation in CI with respect to PTV size or lung density, suggesting that modern planning algorithms can reliably ensure conformity even under extreme low-density conditions and target sizes.

**Figure 6:**
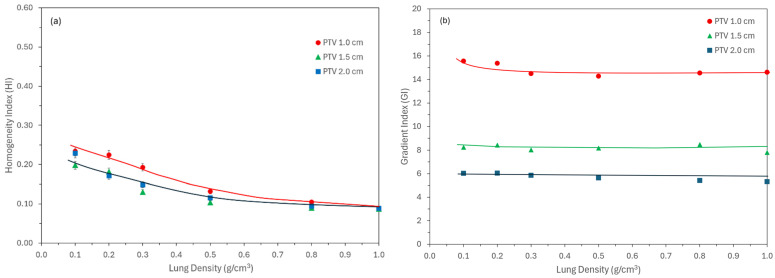
(**a**) Homogeneity index (HI) and (**b**) gradient index (GI) as a function of lung density for three lesions. HI was elevated at lower densities due to increased electron range but plateaued and converged (~0.1) at densities > 0.5 g/cm^3^. Lines are drawn to show the trend in graphs.

The HI that reflects DVH steepness is shown in Figure 6a for all PTV sizes across varying lung densities. At lower densities, increased electron range resulted in higher HI values, indicating poorer dose homogeneity. HI values plateaued around 0.5 g/cm^3^ and converged to ~0.1 at higher densities, independent of lesion size. This behavior differs slightly from multi-institutional SBRT data reported by Das et al. [15], which used a reference HI of 0.2 across sites. Similar variability in HI tails has also been reported in other disease sites, such as prostate cancer [27].

The GI was introduced in ICRU 91 [24], quantifying dose spillage outside of the target volume. Some studies, including Radiation Therapy Oncology Group (RTOG), have used R_50_ instead; however, in this study we followed international guidelines. Figure 6b shows the GI vs. lung density for the three lesions. It is intuitive that for small lesions, where buildup depth is not sufficient (e.g., d_max_ = 1.5 cm in tissue for 6 MV), the GI is much higher. The values are relatively constant to 15, 8.5, and 6 for 1.0, 1.5, and 2.0 cm PTV, respectively. For 1.0 cm PTV, the curve tends to be higher at a lower density, which is reflective of further dose spillage. Extreme lung densities are possible for some patients, especially in deep inspiration where GI will be much higher and attention should be paid.

Large numbers of monitor units (MU) are required in SBRT, particularly for VMAT, and even more so for IMRT (3–5 times that of VMAT). MU per unit dose (MU/Gy) therefore serves as an important measure of beam modulation and plan efficiency. Figure 6 shows MU/Gy plotted against lung density. As noted previously, MU values varied between iterations due to the stochastic nature of optimization; repeated runs were, therefore, performed, and error bars represent this variability. In this study, variability ranged from 1.1% to 7.5%, though in clinical cases values up to 20% have been observed, underscoring the sensitivity of MU to optimization parameters. As expected, MU/Gy increased with decreasing lung density and with smaller PTVs. For larger PTVs, MU/Gy remained relatively constant, whereas for smaller lesions the values plateaued around 0.8 g/cm^3^ density.

## 4. Discussion

Lung SBRT is a mature field with extensive literature on technical considerations and outcomes. While prior reports have described variability in coverage and deviations from international guidelines [23,28], they have not adequately explained the density- and size-dependent nature of these effects. It is well established that dosimetric parameters in lung SBRT strongly influence clinical outcomes [29,30,31]. Our study contributes by quantifying dosimetric metrics (CI, HI, GI, maximum dose, MU/Gy) in small lesions across a range of lung densities, thereby providing a more detailed understanding of how electron transport in low-density media leads to dose spillage. This effect will be more pronounced for higher energies but is not recommended for lung treatments.

It is very difficult to construct experiments on patients as data will be variable. To circumvent this issue, we have performed controlled experiments involving an in silico “pseudo patient”, where all patient anatomy is held constant while varying only two controlled parameters in the planning, e.g., target size and lung density. An important limitation of this work is the focus on small lesions up to 2 cm in diameter. Although these targets are clinically relevant, given their prevalence in screening-detected early-stage lung cancers, future investigations should extend this analysis to larger lesions (>2 cm) to generalize the findings.

**Figure 7:**
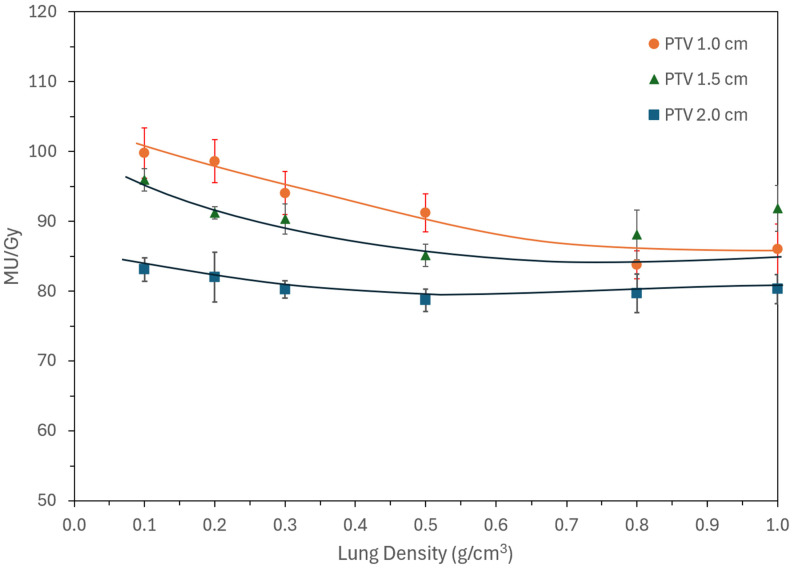
MU/Gy as a function of lung density for three PTV diameters. MU/Gy was higher for smaller targets and lower-density lungs. Error bars reflect variability between repeated optimizations, highlighting the stochastic nature of plan convergence. Lines are drawn to reflect the trend.

Irrespective of density and target size, modern treatment planning systems optimize the conformity as desired, which is shown in Figure 5 and does not vary with either size or density. Figure 6 and Figure 7 provide clinical data for various dosimetric indices with respect to target size and lung density, providing a glimpse of electron transport. For low-density and small lesions, electrons spread out, thus giving higher MU. This reduces to normal values as density and size is increased.

Higher MU requirements are an inherent byproduct of dose spillage in low-density lungs. Elevated MU contributes to increased body dose, which can be approximated by the integral dose (≈∑D_i_·ΔV_i_, where D_i_ is the dose in a bin and ΔV_i_ is the voxel receiving that dose) [32], although the biological implications of integral dose remain poorly defined [33]. It has historically been invoked in comparisons between proton vs. photon therapy and 3D conformal vs. IMRT. Beyond patient exposure, higher MU translates into increased machine workload and wear, underscoring the practical importance of minimizing MU where possible. For plans with similar conformity (CI), those requiring fewer MU should be prioritized [34]. This study advances understanding of lung SBRT dosimetry by quantifying how tumor size, lung density, and key indices (CI, HI, GI, maximum dose, MU/Gy) interact to shape plan quality. Future work will extend these findings across additional planning systems, calculation algorithms, and beam energies to strengthen their generalizability.

## 5. Conclusions

Lung density varies widely among patients and across breathing phases, and this variability strongly influences SBRT dosimetry for small lesions. In this study, we quantified the dependence of key indices (CI, HI, GI, maximum dose, and MU/Gy) on densities ranging from 0.1 to 1.0 g/cm^3^. While conformity (CI ≈ 1.0) was consistently achievable, it came at the cost of substantial dose spillage, particularly for smaller lesions in low-density lungs. GI values ≈ 15, 8, and 6 for 1.0, 1.5, and 2.0 cm lesions, respectively, confirmed this effect and were largely independent of lung density. Monitor unit requirements further reflected the challenges of planning in low-density media: MU and MU/Gy varied between iterations due to the stochastic nature of optimization and were significantly elevated for small lesions and low-density lungs. These findings underscore the importance of accounting for density-dependent physics when interpreting dosimetric indices, comparing outcomes across institutions, and selecting optimal plans. The quantitative benchmarks provided here may inform future studies, planning guidelines, and harmonization efforts in lung SBRT.

## Data Availability

Data are available upon request.

## Abbreviations

The following abbreviations are used in this manuscript:

AAA	Analytical anisotropic algorithm
CI	Conformity index
DVH	Dose—volume histogram
GI	Gradient index
HI	Homogeneity index
HU	Hounsfield unit
LDCT	Low-dose CT
MU	Monitor unit
SABR	Stereotactic ablative radiation therapy
SBRT	Stereotactic body radiation therapy
SSD	Source-to-surface distance
VMAT	Volumetric modulated arc therapy
